# Melatonin Protects MCAO-Induced Neuronal Loss via NR2A Mediated Prosurvival Pathways

**DOI:** 10.3389/fphar.2019.00297

**Published:** 2019-03-29

**Authors:** Fawad Ali Shah, Gongping Liu, Lina T. Al Kury, Alam Zeb, Phil-Ok Koh, Muzaffar Abbas, Tao Li, Xifei Yang, Fang Liu, Yuhua Jiang, Shupeng Li

**Affiliations:** ^1^State Key Laboratory of Oncogenomics, School of Chemical Biology and Biotechnology, Peking University Shenzhen Graduate School, Shenzhen, China; ^2^Riphah Institute of Pharmaceutical Sciences, Riphah International University Islamabad, Islamabad, Pakistan; ^3^Key Laboratory of Ministry of Education of China and Hubei Province for Neurological Disorders, Department of Pathophysiology, School of Basic Medicine and the Collaborative Innovation Center for Brain Science, Tongji Medical College, Huazhong University of Science and Technology, Wuhan, China; ^4^Co-innovation Center of Neuroregeneration, Nantong University, Nantong, China; ^5^College of Natural and Health Sciences, Zayed University, Abu Dhabi, United Arab Emirates; ^6^Department of Anatomy, College of Veterinary Medicine, Research Institute of Life Science, Gyeongsang National University, Jinju, South Korea; ^7^Department of Pharmacy, Capital University of Science and Technology, Islamabad, Pakistan; ^8^Department of Forensic Medicine, School of Medicine, Xi’an Jiaotong University, Xi’an, China; ^9^Centre for Addiction and Mental Health, Campbell Research Institute, Toronto, ON, Canada; ^10^Department of Psychiatry, University of Toronto, Toronto, ON, Canada; ^11^Key Laboratory of Modern Toxicology of Shenzhen, Shenzhen Center for Disease Control and Prevention, Shenzhen, China; ^12^Cancer Centre, The Second Hospital of Shandong University, Jinan, China

**Keywords:** melatonin, ischemic stroke, NMDA receptor, AMPA receptor, PI3K/AKT/GSK3 pathway

## Abstract

Stroke is the significant cause of human mortality and sufferings depending upon race and demographic location. Melatonin is a potent antioxidant that exerts protective effects in differential experimental stroke models. Several mechanisms have been previously suggested for the neuroprotective effects of melatonin in ischemic brain injury. The aim of this study is to investigate whether melatonin treatment affects the glutamate N-methyl-D-aspartate (NMDA) and alpha-amino-3-hydroxy-5-methyl-4-isoxazole propionic acid (AMPA) receptor signaling in cerebral cortex and striatum 24 h after permanent middle cerebral artery occlusion (MCAO). Melatonin (5 mg/kg) attenuated ischemia-induced down regulation of NMDA receptor 2 (NR2a), postsynaptic density-95 (PSD95) and increases NR2a/PSD95 complex association, which further activates the pro-survival PI3K/Akt/GSK3β pathway with mitigated collapsin response mediator protein 2 (CRMP2) phosphorylation. Furthermore, melatonin increases the expression of γ-enolase, a neurotrophic factor in ischemic cortex and striatum, and preserve the expression of presynaptic (synaptophysin and SNAP25) and postsynaptic (p-GluR1845) protein. Our study demonstrated a novel neuroprotective mechanism for melatonin in ischemic brain injury which could be a promising neuroprotective agent for the treatment of ischemic stroke.

## Introduction

Stroke is the second leading cause of death worldwide. Around 5 million dies with another 5 million disabled permanently each year (Goldberg, 2002). Thrombolytics, such as activase and TNKase remained the few available pharmacological choices for clinical application, and which is severely restricted within 3–4.5 h of the onset of ischemia due to potential side effects (Melandri et al., 2009). In the past decades, extensive research work to unknot the complex mechanisms of neuronal death after stroke revealed that Glu accumulation results in the overload of calcium that mainly enter via NMDAR ionotropic channels, eliciting NO activation induced downstream signaling and neuronal death (Scannevin and Huganir, 2000). Numerous NMDAR antagonists have been developed that resulted in repetitive failures in clinical trials (Lai et al., 2014). Further studies suggested that NMDAR mediate contrasting effects depending on its localization, and subunits constitution (Ikonomidou and Turski, 2002). NR2a stimulation predominantly at the synapse induced pro-survival signaling, while NR2b preferably at the extra synaptic areas mediate the excitotoxic death pathways (Hardingham et al., 2002; Liu et al., 2007). These results, although simply explained the controversy, were later compromised by the ambiguous effects of NR2b selective antagonist in clinical trials (Lai et al., 2014). It is speculated that both subunits accounts for essential glutamatergic signaling for physiological function, which severely limit the feasibility of NMDAR antagonist as a therapeutic target in ischemic stroke. Recent efforts have focused on promising neuroprotective agents act downstream of NMDAR, which impedes Glu-mediated neurotoxicity without compromising glutamatergic neurotransmission (Tu et al., 2010).

Among various endogenous and synthetic neuroprotective compounds such as estrogens and progesterone, melatonin is the most extensively studied neuroprotective compound. Melatonin is an indoleamine produced mainly in the pineal gland as well as many other tissues such as retina, gut, and glia cell (Venegas et al., 2012). It is especially effective as a powerful antioxidant, anti-inflammatory agent and a regulator of circadian and seasonal cycles. The amphiphilic character of melatonin enables it to readily cross the BBB, where in the CNS melatonin receptors are widely distributed (Yeleswaram et al., 1997; Chen et al., 2006). Moreover, melatonin has a broad interacting profiles with intracellular proteins including nuclear receptor ROR/RZR, quinone reductase 2 (MT3), and calmodulin (Benitez-King et al., 1993; Cutando et al., 2014). The biological activities of melatonin cannot be attributed to single pathway or receptor; but many targets are involved including transduction pathways. Thus, receptor-dependent and independent actions of melatonin, its low toxicity profile, and clinical safety records make it ideal candidate as neuroprotective agent. In renal ischemia, melatonin exerts nephroprotective effects by attenuating NF-_k_B signaling cascade (Li et al., 2009). In cardiac ischemia, melatonin attenuated oxidative stress by SIRT dependent pathway (Zhai et al., 2017). In this context, the beneficial effects of this indolamine in brain ischemia have been recently reviewed (Ramos et al., 2017). In brain ischemia, several signaling pathways have been proposed for melatonin, including the pro-survival PI3K/AKT, MAPK, oxidative stress related NRF2 and Endothelin-1 (Andrabi et al., 2015). Recently we demonstrated the multimechanistic way by which melatonin modulates the expression of several proteins in ischemic brain (Shah et al., 2018b). However, the exact mechanism linking these molecular changes to excitotoxicity initiated by NMDAR remain unknown.

In this manuscript, we have examined the cascading molecular changes downstream of NMDARs. Our results indicated that melatonin may exert its protective effects by preventing NR2a cleavage via promoting PSD95-NR2a interaction and by increasing γ -enolase/PI3K/AKT/CRMP2 survival pathways.

## Experimental Procedures

### Animals Grouping and Drug Treatment

Adult male Sprague–Dawley rats weighing 200–230g (8–9 weeks) were purchased from Guangdong medical laboratory animal center, China. The experimental animals were housed at Laboratory Animal Research Center, Peking University Shenzhen Graduate School, under 12 h light/12 h dark cycle at 18–22°C and had free access to diet and tap water throughout the study. The experimental procedures were set in such a way to minimize rats suffering. All experimental procedures were carried out according to the protocols approved by Institutional Animal Care and Use Committee of Peking University Shenzhen Graduate School. The rats were randomly divided into 4 groups, each group containing 14 rats: Sham operated control group/Sham; Rats undergoing permanent middle cerebral artery occlusion/MCAO; Melatonin treatment to rats undergoing permanent middle cerebral artery occlusion/Mela+ MCAO; Melatonin treatment to sham operated group/Mela + Sham.

Single dose of melatonin (5 mg/kg, Sigma, St. Louis, MO, United States) or vehicle were administered intraperitoneally 30 min before ischemia. This dose of melatonin showed maximum neuroprotective effects in brain ischemia based upon dose–response studies of melatonin in focal cerebral ischemia (Andrabi et al., 2015). Overall 6 rats died during experimental procedures, three from MCAO, two from Mela+MCAO and one from sham group.

### MCAO Surgery

Middle cerebral artery occlusion was operated as previously described method (Shah et al., 2014, 2016; Sung et al., 2016). Briefly, rats were anesthetized by mixture of xylazine and ketamine (1:3.2, I/P). The right common carotid artery, external carotid artery, and internal carotid artery were exposed through a midline cervical incision. The occipital artery, superior thyroid arteries were knotted with (6/0) silk and subsequently cut. The external carotid artery was knotted and a nylon filament (3/0) with blunted rounded tip about 30 mm in length was inserted from the external carotid artery into internal carotid artery and advanced further into the origin of the middle cerebral artery, whereby some resistance to the movement of nylon occurred. The sham operated animals were subjected to the same procedures except the insertion of filament. 24 h after the onset of permanent occlusion, animals were decapitated with chloroform, and brain tissues were collected. The major limitation associated with this model is subarachnoid hemorrhage due to vessel rupturing and hyperthermia.

### Neurobehavioral Test

Twenty eight-point composite neuroscore was used to evaluate the sensorimotor deficits based on a number of tests including (1) circling, (2) motility, (3) general condition, (4) righting reflex when placed on its back, (5) paw placement on table top, (6) ability to pull itself up on a horizontal bar, (7) climbing on an inclined platform, (8) grip strength, (9) contralateral reflex, (10) contralateral rotation when held by the base of its tail, and (11) visual forepaw reaching. A cumulative score of 28 indicated healthy functioning while 0 for severe neurological impairment.

### TTC Staining

Rats after permanent occlusion were decapitated under anesthesia at the end of 24 h period. Brain tissue were carefully removed and washed with cold PBS. 3–4 mm-thick coronary sections were cut by using sharp blade from frontal lobe. These coronal slices were incubated in 2% 2,3,5-triphenyltetrazolium chloride (TTC) (solution was prepared in PBS) for 10–20 min, until a thorough demarcation was observed for MCAO operated rats, while sham operated rats were stained deep red (Shah et al., 2018a). The coronal sections were then fixed in 4% paraformaldehyde and photographed. The % infarct area was then measured by utilizing ImageJ, computer based program. To compensate for brain edema, the corrected brain infarction was calculated as fallow: Corrected infarct area = left hemisphere area – (right hemisphere area – infarct area).

### Western Blot

For the Western blot analysis, the samples were dissolved in lysis buffer containing (1M Tris–HCI, 5M sodium chloride, 0.5% sodium deoxycholate, 10% sodium dodecyl sulfate, 1% sodium azide, 10% NP-40) as described previously (Shah et al., 2014) The homogenate was sonicated and centrifuged at 15,000 rpm for 20 min at 4°C and protein concentration was determined by BCA kit (Pierce, Rockford, IL, United States) according to the guidelines provided by manufacturer. Equal amount of protein i.e., (30 μg per sample) were electrophoresed on 10% SDS-PAGE gels followed by immunoblotting for transferring the protein to PVDF membranes (Millipore, Billerica, MA, United States). To minimize the non-specific antibody binding, the PVDF was blocked with skim milk for 1 h at room temperature. PVDF was washed in TBST and then incubated with primary antibodies overnight at 4°C. The membranes were then incubated with appropriate secondary antibody, and protein band were detected using an ECL detection reagent according to the manufacturer’s instructions (Amersham Pharmacia Biotech, Piscataway, NJ, United States).

The antibodies used include anti-NR2a (SC-1468), anti-PSD95 (SC-71933), anti-GluR1 (SC-55509), anti-SNAP25 (SC-7538), anti-synaptophysin (SC-17750), anti-γ-enolase (SC-71046), anti-Ppi3k (SC-293115), Anti-CRMP2 (SC-376739), anti-p-AKT Ser473 (SC-7985), anti-p-GSK3β Ser9 (SC-11757), and anti-β-Actin (SC-130656) from (Santa Cruz, Biotechnology, CA, United States). Anti-p-CRMP2 Thr 514 (Cat#9397) and anti-p- GluR1serine 845 (Cat#4511), was obtained from CST and were used at dilution of 1:1000.

### Tissue Collection for Morphology

We used (*n* = 7 rats/group) for tissue analysis. The brain after collection form different experimental groups was fixed in 4% paraformaldehyde. The brains were then imbedded in paraffin, and 4 μm coronary sections were made by using rotary microtome.

### Cresyl Violet Staining

Tissue sections on coated slides were de-paraffinized with three treatments of absolute xylene and rehydrated with graded ethyl alcohol. The slides were rinsed with distilled water and immersed in 0.01 M PBS for 10 min. 0.5% (w/v) cresyl violet acetate (Sigma) was dissolved in distilled water, and a few drops of glacial acetic acid were added to the solution just before use. Brain sections were stained with cresyl violet solution for approximately 20 min. The slides were rinsed with distilled water and differentiated in ethyl alcohol (70, 95, and 100%). The slides were cleared with xylene and were mounted with glass cover slip. The stained images were pictured with light microscope (Olympus, Japan), using 20× magnification scale, saved as TIF files and analyzed by ImageJ, computer based program. The number of images per slide was five per group. The TIF images was optimized to the same threshold intensity for all groups for pyknotic, red and ghost neurons.

### Tunel Staining

Tunnel staining was performed according to manufacturer’s protocol the DNA Fragmentation Detection Kit (Oncogene Research Products, Cambridge, MA, United States). The de-paraffinized sections were permeated with proteinase K followed by inactivation of peroxidases. The slides were incubated subsequently in equilibration buffer and with TdT labeling reaction mixture at 37°C for 1.5 h. This reaction was stopped by stopper buffer. The sections were washed with PBS and stained in DAB solution, washed with distilled water, dehydrated in graded ethanol (70, 95, and 100%), and fixed in xylene and cover-slipped by mounting medium. Immunohistochemical TIF images were captured with a light microscope, using 20x magnification scale taking five images per slide. ImageJ software was used to quantitatively determine TUNEL positive cells in the frontal cortex and striatum areas. Briefly, TIF images was optimized to the same threshold intensity for all groups and was expressed as the relative TUNEL neuronal cells/ section of the samples relative to the control.

### Immunofluorescence Analysis

After de-paraffinization of sections, the slides were autoclaved in 0.1M sodium citrate pH 6 for antigen retrieval step. The slides were allowed to cool and washed with PBS twice times. Slides were incubated with 5% normal serum depending upon the source of secondary antibody used. The slides were incubated with primary antibodies at 4°C overnight (PSD95, NR2a, synaptophysin, γ-enolase, p-PI3K) from Santa Cruz Biotechnology, and rabbit monoclonal CRMP2, from cell signaling overnight at 1:100 dilution. Next morning, after washing with PBS, fluorescent labeled secondary antibodies (Santa Cruz Biotechnology) were used for signal amplification in dark chamber, followed by mounted with UltraCruz mounting medium (Santa Cruz Biotechnology). The slides were pictured with Confocal scanning microscopes (Flouview FV 1000, Olympus, Japan) using 40× magnification scale, and analyzed by ImageJ, computer based program. ImageJ software was used to quantitatively determine fluorescence intensity of the same region (frontal cortex and striatum) for all groups by optimizing background of image according to the threshold intensity and analyzes the immunofluorescence intensity at the same threshold intensity for all groups and was expressed as the relative integrated density of the samples relative to the control.

### Statistical Analysis

Western blot bands and morphological data was analyzed using ImageJ software (Image J 1.30 ^1^). Data were presented as means ± SEM. Data were analyzed by two ways ANOVA followed by *post hoc* Bonferroni Multiple Comparison test using graph-pad prism-5 software. Moreover, TTC staining and neurological deficit scores were analyzed by Student’s *t* test. Symbols ^∗^ or ^#^ represent significant difference values *p* < 0.05, ^∗∗^ or ^##^ represent *p* < 0.01 and ^∗∗∗^ or ^###^ represent *p* < 0.001 values for significant differences. Symbols ^∗^ shows significant difference relative to Sham, while ^#^ shows significant difference relative to MCAO.

## Results

### Effect of Melatonin on Ischemic Induced Neurodegeneration and Apoptosis

**FIGURE 1:**
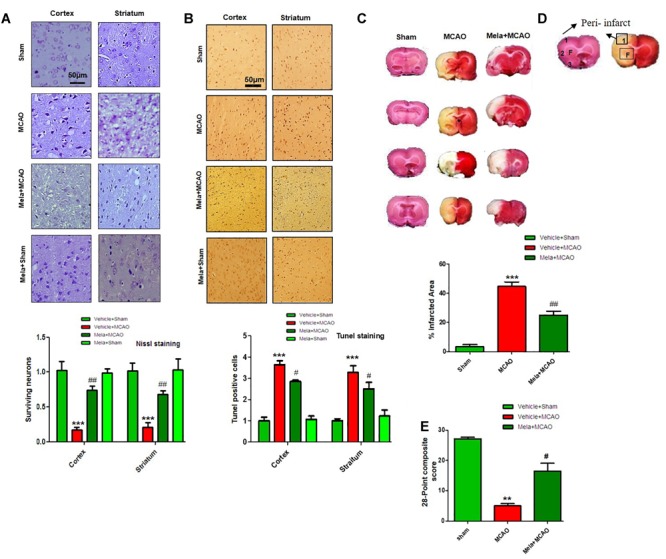
Effect of melatonin on neurological scores, brain infarction and neurodegeneration. **(A)** Effect of melatonin on Nissl staining. Representative photomicrograph of cresyl staining showing the extent of surviving neurons in cortex and striatum; (*n* = 7 animals/group). The number of experiments performed = 3 and data presented is relative to sham. Magnification 20×, scale bar = 50 μm. **(B)** Tunel histochemistry with representative photos showed apoptotic cells (scale bar = 50 μm, magnification 20×). Images are representative of experiments performed 3 times with *n* = 7 per group. MCAO causes significant neuronal apoptosis; while treatment with melatonin attenuated apoptotic damage. **(C)** Brain coronal sections were stained with TTC, which distinguishes between ischemic and non-ischemic areas. Student’s *t*-test was used for analysis, (*n* = 7/group. ^∗∗∗^ indicates *p* < 0.001, while symbols ^∗∗^ and ^##^ represent *p* < 0.01 significantly different; ^#^ indicates *p* < 0.05. Symbols ^∗^ shows significant difference relative to sham control while ^#^ shows significant difference relative to MCAO. **(D)** Coronal sections separated by frontal cortex (1), parietal cortex and insular cortex (2), and periform cortex (3). The analyzed region of interests indicated by square 1 and F. **(E)** 28 points composite scoring. Mela+MCAO rats had significantly less severe neurological deficits (^#^*p* < 0.05) than MCAO rats had. Mann-Whitney (non-parametric test) is applied and neurological score data is presented as the median and range (*n* = 10–14/group).

During MCAO, robust neuronal changes occurred more frequently in the core brain where impaired blood supply caused irreversible cell death. Nissl staining was performed in frontal cortex and striatum areas to examine the neuroprotective effects of melatonin and to differentiate necrotic neurons from intact ones. Compared to sham operated animals, a substantial difference was observed after 24 h of permanent ischemia, which showed aberrant morphological features, such as alteration in neuronal size and shape (swelling and scalloped angular nature), alteration in color staining (cytoplasmic eosinophilia/pyknosis and basophilic nature of nucleus) and vacuolation (Figure 1A). A significant larger number of morphological intact cells could be found in melatonin treated groups (Figure 1A).

Neuronal apoptosis could be seen after MCAO in the ischemic penumbra or peri-infarct zone, where blood flow is less severely reduced. Tunel staining was performed in the frontal cortex and striatum areas to examine the neuroprotective role of melatonin on cell apoptosis. As expected, a significant number of Tunel-positive cells was observed in the infarcted neocortex and caudate putamen of striatum in MCAO rats (Figure 1B,D), which are compactly stained and accompanied with fragmented apoptotic bodies, a phenomenon that could be significantly decreased by melatonin treatment (Figure 1B). Further calculations of the corrected percent infarcted area in MCAO and Mela+MCAO were about to be 45.92 ± 4.3% and 28.71 ± 3.9% (Figure 1C). Neurological scores were determined 24 h after the onset of permanent MCAO. Rats subjected to MCAO exhibited severe neurological deficits. Further analysis of 28 points composite scoring indicated that sensorimotor functions were hampered in MCAO rats (Figure 1E), which was tremendously reversed by melatonin treatment (Figure 1E).

### Effect of Melatonin on Glutamatergic Receptors Following Permanent MCAO

**FIGURE 2:**
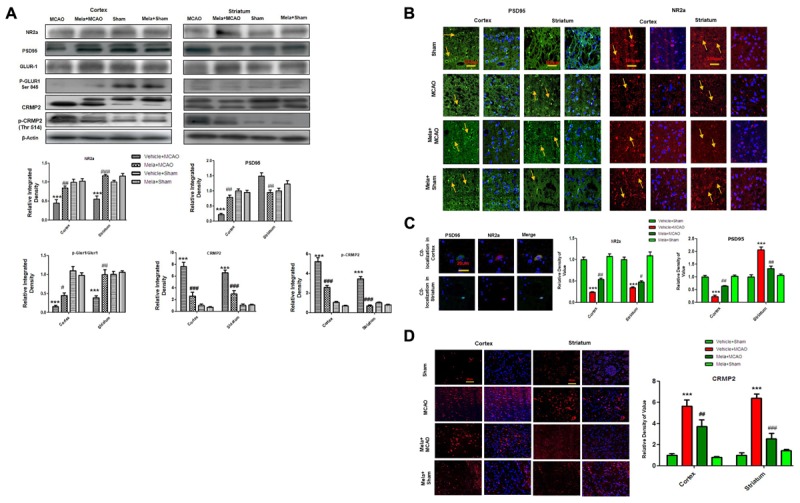
Effect of melatonin on glutamate (Glu) receptor subunits. **(A)** Western blot analysis of NR2a, PSD95, GluR1, p-GluR1 (Serine 845), CRMP2, p-CRMP2 (Thr 514) from sham-operated control group, vehicle-treated MCAO, melatonin-treated MCAO animals and melatonin treated sham group (*n* = 7 rats per group). The bands were quantified using ImageJ, analyzed by graph-pad prism-5 software. β-actin was used as control. Densitometric analysis was expressed in arbitrary units as the mean ± SEM for the indicated proteins. **(B)** Immunofluorescence reactivity of PSD95 and NR2a; (*n* = 7 rats/group). The above data is representation of 3 numbers of experiments. Scale bar = 100 μm and magnification 40×. PSD95 and NR2a show cytoplasmic localization and was visualized by FITC and TRITC. **(C)** Double immunofluorescence of NR2a and PSD95 in ischemic brain. PSD95 was visualized by FITC and NR2a by TRITC. Scale bar = 20 μm and magnification 40×. **(D)** Immunofluorescence reactivity of CRMP2; (*n* = 7 rats/group). The above data is representation of 3 numbers of experiments. Scale bar = 50 μm. CRMP2 was visualized by Alexa-Flour 594. Symbols ^#^ represent significant difference values *p* < 0.05, while symbol ^##^ represent *p* < 0.01 values for significant differences, symbol ^∗∗∗^ and ^###^ shows *p* < 0.001. Symbols ^∗^ shows significant difference relative to sham control while ^#^ shows significant difference relative to MCAO.

Accumulated evidences suggested that NMDAR mediated excitotoxicity accounted for the delayed and progressive neuronal damage following ischemia. The underlying molecular and cellular mechanisms remained the center for stroke research in the last decade (Hoyte et al., 2004; Martin and Wang, 2010; Wu and Tymianski, 2018). Synaptic Glu activity mainly activated NR2a-containing NMDAR, leading to the activation of pro- survival signaling proteins including Akt, ERK, and CREB (Thomas and Huganir, 2004; Terasaki et al., 2010). We, therefore, studied the expression of NR2a subunit following permanent MCAO and the effect of melatonin treatment. Our results showed that full length NR2a protein level was significantly decreased in ischemic conditions as compared to sham operated group (Figure 2A), while the expression changes could be reversed on melatonin treatment both in cortex and striatum, respectively (Figure 2A). The above results were further confirmed using immunofluorescent staining, which revealed NR2a immunostaining dramatically decreased in MCAO operated group (Figure 2B), whereas a strong NR2a immunoreactivity was observed in cortex and striatum of Mela+MCAO group (Figure 2B). These results further support the previous observations, in which MCAO induced decreased expression of NR2a is implicated with reduced downward signaling cascade (Chen et al., 2008; Gascon et al., 2008; Yuen et al., 2008).

Calpain activation leads to the degradation and decreased expression of full length NR2a in MCAO (Gascon et al., 2008; Yuen et al., 2008) an effect that could be attenuated by increased interaction of PSD95/NR2a. We then investigated whether melatonin affected PSD95/NR2a interaction. Similar expression patterns of PSD95 and NR2a were found in cortex across treatment groups (Figure 2A,B), and their co-localization could be defined in sham operated animals (Figure 2C). However, this interaction was tremendously reduced in the ischemic brain, showing parallel decrease of both PSD95 and NR2a in cortex (Figure 2A–C). Together, these results suggested that melatonin may exert its neuroprotective effects by attenuated the cleavage of full length NR2a.

CRMP2 displayed proteolytic cleavage after MCAO. Previous studies demonstrated close association of NMDA receptor with CRMP2 (Al-Hallaq et al., 2007) because such mechanisms regulate the trafficking, expression and internalization of NMDAR receptor. We then examined the potential effects of CRMP2 on melatonin treatment. As shown in (Figure 2A) CRMP2 exhibited a migration pattern of cleaved bands (Figure 2A), with the intact mass of CRMP2 at 66kD degrading to 62 and 55kD protein in MCAO group (Zhang et al., 2007). Melatonin treatment attenuated this cleavage. Similarly, immunofluorescence results further validated the expression of these CRMP2 protein (*p* < 0.001, Figure 2D). Moreover, the phosphorylated form of CRMP2 (CRMP2 Thr514) also showed higher expression in MCAO (Figure 2A, *p* < 0.001).

AMPA receptor activation is triggered by Glu accumulation following cerebral ischemia. In line with previous reports, we observed a significantly reduced expression of AMPAR (GluR-1) receptor following cerebral ischemia (Figure 2A) compare to sham operated group. Melatonin treatment reversed the effect of ischemia on AMPA receptors (Figure 2A). Similarly, the phosphorylation levels of AMPA (GluR1) at serine 845 were studied and the results showed a marked decrease following MCAO, as compared to sham operated control (Figure 2A). Melatonin treatment significantly reversed the changes in the p-AMPA in cortical and striatal tissues (Figure 2A).

### Melatonin Prevents Synaptic Dysfunction by Preserving Synaptic Protein Following Cerebral Ischemia

**FIGURE 3:**
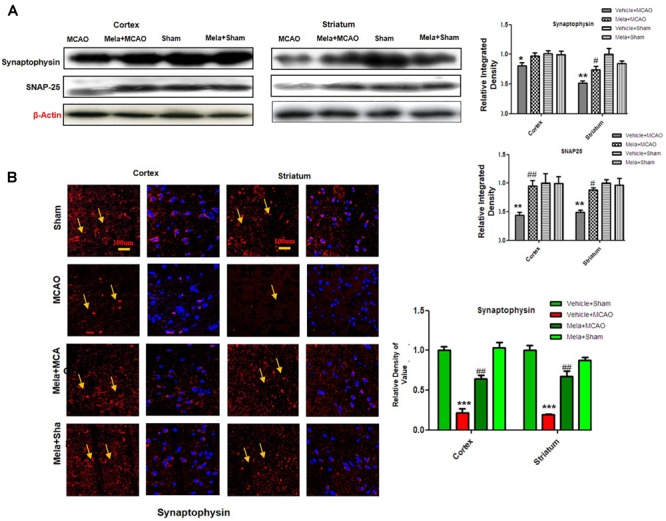
Melatonin protects synaptic protein from ischemic injury. **(A)** Western blot analysis of SNAP-25 and synaptophysin from ischemic cortex and striatum of vehicle-treated MCAO, melatonin-treated MCAO animals, sham-operated control group, and melatonin treated sham group (*n* = 7 rats per group). The bands were quantified using ImageJ, analyzed by graph-pad prism-5 software. β-actin was used as control. Densitometric analysis was expressed in arbitrary units as the mean ± SEM for the indicated proteins. **(B)** Immunofluorescence reactivity of synaptophysin; (*n* = 7 rats/group). The above data is representation of 3 numbers of experiments. Scale bar = 100 μm and magnification 40×. Synaptophysin was visualized by Alexa-Flour 594. Symbols ^∗^ or ^#^ represent significant difference values *p* < 0.05, while symbols ^∗∗^ and ^##^ represent *p* < 0.01 values for significant differences, symbol ^∗∗∗^ shows *p* < 0.001. Symbols ^∗^ shows significant difference relative to sham control while ^#^ shows significant difference relative to MCAO.

Synaptic plasticity and synaptogenesis are highly desirable cellular processes for functional restoration after ischemic stroke. It has been demonstrated that SNAP-25, and synaptophysin acts as synaptic marker for neuronal differentiation (Bailey and Lahiri, 2006; Goddard et al., 2008). To examine the neuroprotective effect of melatonin on synaptic proteins following cerebral ischemia, the expression of synaptophysin, and SNAP-25 were studied. The results showed that synaptophysin and SNAP-25 expression were significantly decreased in MCAO groups, while melatonin treatment reversed the deleterious effect on these proteins (Figure 3A). Moreover, immunofluorescence showed a strong synaptophysin immunoreactivity in the cortex and striatum of the sham operated control group, which was considerably diminished following 24 h after MCAO but recovered in melatonin treated group (Figure 3B).

### Melatonin Promote γ-Enolase/PI3K/AKT/GSK-3β/CRMP2 Pathway

**FIGURE 4:**
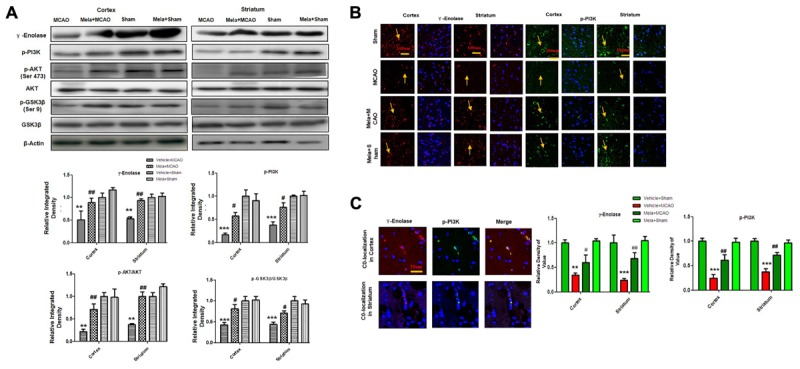
Melatonin induces neuroprotection via PI3K/AKT/GSK-3β/CRMP2 pathway. **(A)** Western blot analysis of γ–enolase, p-PI3K, p-Akt, Akt, p-GSK-3β, GSK-3β, CRMP2, p-CRMP2 from cortical, and striatal tissues (*n* = 7 rats/group). The bands were quantified using ImageJ, analyzed by graph-pad prism-5 software. β-actin was used as control. Densitometric analysis was expressed in arbitrary units as the mean ± SEM for the indicated proteins. **(B)** Immunofluorescence reactivity of γ-enolase and p-PI3K; (*n* = 7 rats/group). The above data is representation of 3 numbers of experiments. Scale bar = 100 μm and magnification 40×**(A)** γ-enolase **(B)** p-PI3K show cytoplasmic localization and was visualized by TRITC and FITC. **(C)** Double immunofluorescence of γ-enolase and p-PI3K in ischemic brain. The cortical and subcortical sections show correspondingly decreased expression of γ-enolase and p-PI3K after 24 h of permanent ischemia. γ-enolase visualized by TRITC and p-PI3K was by FITC. Symbol ^#^ represent significant difference values *p* < 0.05, while symbols ^∗∗^ and ^##^ represent *p* < 0.01 values for significant differences, symbol ^∗∗∗^ shows *p* < 0.001. Symbol ^∗^ shows significant difference relative to sham control while ^#^ shows significant difference relative to MCAO.

Apart from its effects on NR2a expression and NR2a/PSD95 interaction, melatonin can promote a variety of neurotrophic factors. Previous reports demonstrated that γ-enolase enhances neuronal survival, differentiation, and neurite regeneration via activating the PI3K/Akt and other related signaling pathways (Hafner et al., 2012). To examine the potential effect of melatonin on these neuronal survival and regenerative related molecules, we performed western blot analysis. Our results showed that γ-enolase expression decreased during brain ischemia, and melatonin treatment recovered the protein expression level (Figure 4A,B). Moreover, a strong immunoreactivity was noticed in cortex and striatum of sham operated group and Mela + MCAO operated group for γ-enolase and p-PI3K (Figure 4A,B), while this immunostaining dramatically decreased in MCAO operated group (Figure 4B). γ-enolase and p-PI3K were co-localized by double immunofluorescence (Figure 4C). Together, these results demonstrated that melatonin might exert its neuroprotective effects via increasing γ-enolase/PI3K/Akt pathway, with Akt at serine 473 working as survival kinases which prevent apoptosis by phosphorylating its downstream target glycogen synthase kinase-3β (GSK-3β at serine 9).

## Discussion

In the present study, we have demonstrated that melatonin attenuates MCAO-induced apoptosis and neurodegeneration both in cortical and striatal tissue. The extent of injury caused by MCAO depends upon the duration of occlusion. The permanent MCAO produces the most uniform type of infarction which frequently involves the neocortex and striatum (Popp et al., 2009). Blood flow to striatum is lower than cortex; due to which striatum is severely hit by ischemic stroke (Lo et al., 2003). This is because blood supply to the striatum is provided by tiny unidirectional vessels from MCA and there is no collateral connection to this area form surrounding vasculature (Fluri et al., 2015). Our study showed that melatonin treatment protected NR2A from ischemic cleavage via increasing its coupling to PSD95, which further promoted NR2A mediated pro-survival signaling of γ-enolase/pI3K/Akt/GSK3β/ CRMP2 pathway. Finally, we also demonstrated synapto-protective effect of melatonin and suggested that melatonin attenuates MCAO induced oxidative stress possibly by preserving synaptic structure of protein. Moreover, stroke therapy academy industrial roundtable (STAIR) recommends neuroprotective agents should protect gray and white matter from ischemic torment (Fisher et al., 2009).

The role of NMDA subunits in ischemic brain injury is intensively debated due to contradicting results. Previous results pointed out the major shortfalls accompanied with Glu antagonism which showed repetitive clinical trial failures (Ikonomidou and Turski, 2002; Hoyte et al., 2004). It is coequally believed that NR2a subunit of NMDA has pro survival while NR2b is linked to neuronal apoptosis irrespective of synaptic location (Liu et al., 2007; Chen et al., 2008). Further reports suggested potential therapeutics mechanisms by selectively targeting proteins implicated in trafficking, expression and internalization of NMDAR receptor, without altering the intrinsic properties of receptor (Kemp and McKernan, 2002). Calpain is activated by ischemic injury, which degrades a large array of molecules including NMDA receptor subunits and are implicated in the pathogenesis of MCAO (Kemp and McKernan, 2002; Simpkins et al., 2003). In agreement to previous reports, we shown here that 24 h of cerebral ischemia down regulated NR2a and melatonin treatment counteract this proteolysis. Furthermore, NR2a is protected from calpain degradation by PSD95 shielding. In cortex, we observed down regulation of PSD95 which might expose NR2a to calpain mediated cleavage, whereas melatonin treatment restored PSD95 expression and maintained the protective interaction of PSD95 with NR2a.

AMPA receptor subunits (GluR1, GluR2, GluR3, and GluR4) also play a vital role in Glu induced excitotoxicity. AMPA receptor activity is controlled by several mechanisms including phosphorylation and calpain mediated cleavage of GluR1 subunit (Yuen et al., 2007). In line with previous reports we observed down regulation of GluR1 in ischemic cortex and striatum in our ischemic model. Phosphorylation at serine 845 is involved in the translocation of AMPA to neuronal membrane (Esteban et al., 2003). Such AMPAR actions boost synaptic plasticity for learning and memory. Our results that melatonin treatment recovered GluR1 serine 845 phosphorylation could increase and stabilize AMPAR membrane surface expression, which could possibly be the underling neuroprotective mechanism in memory impairment model.

Ischemic stroke rigorously disrupts synaptic networks, while some reports suggested synaptic remolding after ischemic damage with correspondingly increased synapse formation (Hammond et al., 1994; Jourdain et al., 2002). Synaptophysin and SNAP-25 level was compromised in ischemic brain injury compare to melatonin treated groups. Moreover, PSD95 showed hyper expression in permanent ischemic injury for reasons explained previously (Martone et al., 1999; Hu et al., 2000; Kovalenko et al., 2006). Both PSD95 and synaptophysin, promotes synaptic plasticity, and synaptogenesis. Moreover, some studies demonstrated altered expression of PSD95 to cognitive impairment (Yan et al., 2013). The expressions of both these proteins are markedly attenuated in ischemic cortex and melatonin treatment rescue these proteins from ischemic torment.

Impairment of the PI3K/Akt signaling pathway is implicated in major neurodegenerative diseases including ischemic stroke. γ-enolase, also called neuronal specific enolases (NSE), an important molecule to directly assesses neuronal damage and repair, has been shown to control neuronal survival, differentiation, and neurite regeneration by activating the PI3K/Akt and MAPK/ERK signaling pathways (Lee et al., 2004, 2005). γ-enolase mediated PI3K activation, also regulates RhoA kinase, a key regulator of actin cytoskeleton organization, and may influence both neurodegeneration and neuroprotection depending on the signal strength. Our results showed attenuated expression of γ-enolase and PI3K in ischemic brain, whereas melatonin treatment enhanced γ-enolase/PI3K/Akt signaling pathway, suggesting the pro-survival signaling possibly involving NR2a. These results are in agreement with previous reported studies (Shehadah et al., 2010; Gim et al., 2015).

CRMP2 is associated with axonal growth as it adds essential microtubule filaments. The biological role of CRMP2 and its phosphorylated form CRMP2 (Thr514) in ischemic brain injury remained unknown. Contradicting reports exist with some studies suggesting hyper-expression of CRMP2 (Indraswari et al., 2009; Shah et al., 2014) while others observed attenuated expression in ischemic brain injury. During brain injury, CRMP2 (66 kDa) was degraded into 55 kDa BDP by calpain mediated proteolysis (Yoshimura et al., 2005; Yang et al., 2016). Distinct from the previous reports of reduced p-CRMP2 expression in neonatal 6-h focal hypoxic ischemic model (Sato et al., 2011; Yang et al., 2016), our results of higher expression of p-CRMP2 are in according with the increased activity of GSK-3β and can be reversed by phosphorylating GSK-3β at Ser-9. Indeed, melatonin restores the expression of PI3-kinase and inactivates Akt via phosphorylating the sites at Thr-308 and Ser-473. This leads to GSK-3β inactivation via Ser-9 phosphorylation, supporting previous results that GSK3-β is negative regulator of axonal growth by modulating CRMP2 activity (Yoshimura et al., 2005; Gim et al., 2013). Thus, melatonin preserved the integrity of CRMP2 and ameliorated the neuropathological changes elicited via CRMP2 phosphorylation at Thr 514 (Takata et al., 2009; Gim et al., 2013). Finally, we have also observed consistent increase in the expression of synaptophysin (axonal marker) by melatonin, which further suggests the protective effect of melatonin on axonal growth.

**FIGURE 5:**
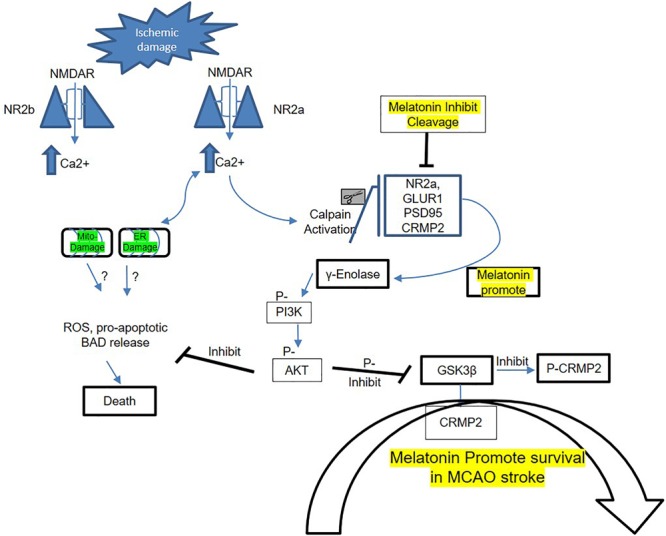
Proposed schematic presentation of melatonin in MCAO stroke. A schematic diagram of suggested neuroprotective mechanism of melatonin in MCAO stroke brain. This diagram shows the possible mechanism by which melatonin prevent MCAO-induced neuronal damage, synaptic deficit, and neurodegeneration through Enolase/PI3K/AKT/CRMP2 pathway.

In conclusion, our results demonstrated that melatonin modulated ischemic injury-induced glutamatergic impairment, synaptic dysfunction, apoptotic marker, and neurodegeneration in ischemic brain. We have shown for the first time (as per our information and literature survey) the aberrant expression of NMDA and AMPA glutamatergic receptor in striatum. Notably, treating rats with melatonin augmented the neurotrophic activity of enolase, which produces neuroprotection by downstream targets (pI3K/Akt/GSK3β/CRMP2). The suggested mechanistic approach of melatonin in MCAO-induced model to attenuate synaptic dysfunction and to mediate NR2a dependent survival pathway is summarized in Figure 5.

## Data Availability

All data generated or analyzed during this study are included in this published article.

## Ethics Statement

All experimental procedures were carried out according to the protocols approved by Institutional Animal Care and Use Committee of Peking University Shenzhen Graduate School.

## Author Contributions

FS and GL managed the experimental work. FS and GL performed surgery, western blot, morphological experiments, and data analysis. AZ, P-OK, MA, LA, FL, TL, XY, YJ, and SL supported the study, designed study, and wrote the manuscript. YJ and SL are the corresponding authors, reviewed and approved the manuscript and held all the responsibilities related to this manuscript. All authors reviewed and approved the manuscript.

## Conflict of Interest Statement

The authors declare that the research was conducted in the absence of any commercial or financial relationships that could be construed as a potential conflict of interest.

## Footnotes

1 https://imagej.nih.gov/ij/

## Abbreviations

BBB: blood brain barrier
BCA: bicinchoninic acid
BDP: break down products
CST: cell signaling technology
DAB, 3: 3′-Diaminobenzidine tetra hydrochloride
Glu: glutamate
GSK-3β: glycogen synthase kinase-3β
MCAO: middle cerebral artery occlusion
NMDAR: N-methyl-d-aspartate receptor
NO: nitric oxide
PVDF: poly-vinylidene fluoride
SEM: standard error of mean
SNAP-25: synaptosomal associated protein 25
TBST: Tris-buffered saline containing 0.1% Tween-20
TdT: terminal deoxynucleotidyl transferase
TTC: quinone reductase
2 MT3: 2,3,5-triphenyltetrazolium chloride
TUNEL: Terminal deoxynucleotidyl transferase dUTP nick end labeling
