# Corrigendum: Melatonin Protects MCAO-Induced Neuronal Loss *via* NR2A Mediated Prosurvival Pathways

**DOI:** 10.3389/fphar.2019.00702

**Published:** 2019-06-21

**Authors:** Fawad Ali Shah, Gongping Liu, Lina T. Al Kury, Alam Zeb, Phil-Ok Koh, Muzaffar Abbas, Tao Li, Xifei Yang, Fang Liu, Yuhua Jiang, Shupeng Li

**Affiliations:** ^1^State Key Laboratory of Oncogenomics, School of Chemical Biology and Biotechnology, Peking University Shenzhen Graduate School, Shenzhen, China; ^2^Riphah Institute of Pharmaceutical Sciences, Riphah International University Islamabad, Islamabad, Pakistan; ^3^Key Laboratory of Ministry of Education of China and Hubei Province for Neurological Disorders, Department of Pathophysiology, School of Basic Medicine and the Collaborative Innovation Center for Brain Science, Tongji Medical College, Huazhong University of Science and Technology, Wuhan, China; ^4^Co-innovation Center of Neuroregeneration, Nantong University, Nantong, China; ^5^College of Natural and Health Sciences, Zayed University, Abu Dhabi, United Arab Emirates; ^6^Department of Anatomy, College of Veterinary Medicine, Research Institute of Life Science, Gyeongsang National University, Jinju, South Korea; ^7^Department of Pharmacy, Capital University of Science and Technology, Islamabad, Pakistan; ^8^Department of Forensic Medicine, School of Medicine, Xi’an Jiaotong University, Xi’an, China; ^9^Centre for Addiction and Mental Health, Campbell Research Institute, Toronto, ON, Canada; ^10^Department of Psychiatry, University of Toronto, Toronto, ON, Canada; ^11^Key Laboratory of Modern Toxicology of Shenzhen, Shenzhen Center for Disease Control and Prevention, Shenzhen, China; ^12^Cancer Centre, The Second Hospital of Shandong University, Jinan, China

**Keywords:** melatonin, ischemic stroke, NMDA receptor, AMPA receptor, PI3K/AKT/GSK3 pathway

**Phil-Ok Koh** was not included as an author in the published article. The corrected Author Contributions Statement appears below. The authors apologize for this error and state that this does not change the scientific conclusions of the article in any way. The original article has been updated.

## Author Contributions

FA and GL managed the experimental work. FA and GL performed surgery, western blot, morphological experiments; performed data analysis. AZ, P-OK, MA, LA, FL, TL, XY, YJ and SL supported the study, designed study, and wrote the manuscript. YJ and SL are the corresponding authors, reviewed and approved the manuscript and held all the responsibilities related to this manuscript. All authors reviewed and approved the manuscript.

